# Generalized Structural Equation Modeling (GSEM) in prediction of injury leading to motorcyclist hospitalization: a case-control study 

**Published:** 2019-08

**Authors:** Shila Hasanzadeh, Mohammad AsghariJafarabadi, Homyoun Sadeghi-Bazargani

**Affiliations:** ^*a*^Department of Statistics and Epidemiology, Faculty of Health, Tabriz University of Medical Sciences, Tabriz, Iran.; ^*b*^Associate Professor in Statistics and Epidemiology, Department of Statistics and Epidemiology, Faculty of Health, Tabriz University of Medical Sciences, Tabriz, Iran.; ^*c*^Associate Professor in Epidemiology, Road Traffic Injury Research Center, Tabriz University of Medical Sciences, Tabriz, Iran.

## Abstract

**Background:**

Generalized Structural Equation Modeling (GSEM) in prediction of injury leading to motorcyclist hospitalization: a case-control study Abstract Background and Objectives: Given that the SEM model includes both structural and measurement models, It is also a method for the exact test of theoretical models and is very useful for causal modeling. The purpose of the present study is to investigate the predictors of injuries caused the hospitalization of motorcyclists using a Generalized Structural Equation Modeling (GSEM) with mediator in a case-control study.

**Methods:**

In this case-control study, 300 cases and 156 controls were selected from 150 clusters using a cluster random sampling in Tabriz, Iran. Using of motorcycle-riding behavior questionnaire (MRBQ), Attention-deficit/hyperactivity disorder (ADHD). The GSEM model was used to examine the linear direct and indirect linear relationships of variables in the conceptual model and considering the binary response variable of the model. Also, MRBQ were considered as a mediator variable for the underlying and ADHD and Data analysis was performed by STATA14 software.

**Results:**

The predictors of injury were: MRBQ, ADHD and Demographic, The results indicated significant linear and direct relationships between odds of injury and cell phone answering (OR= 2.22, P= 0.010), hyper active child (OR= 1.65, P= 0.057), dark hour riding (OR= 1.01, P= 0.001) and MRBQ (OR= 1.27, P= 0.092), while significant inverse relation between injury and being married(OR= 0.43, P= 0.002), academic education (OR= 0.29, P= 0.001).

**Conclusions:**

Based on the results GSEM model, Due to the significance of the variables, having intervention programs, on the ADHD, and those who answer their cell phones while driving, and dark hour riding is highly recommended.

**Keywords:**

SEM, GSEM, Traffic, Injury, MRBQ, ADHD

